# Complete mitochondrial genome of the Japanese field vole *microtus montebelli* (Milne-Edwards, 1872) (Rodentia: Arvicolinae)

**DOI:** 10.1080/23802359.2021.1917315

**Published:** 2021-08-25

**Authors:** Atsushi Sogabe, Chie Murano, Ryota Morii, Hiroshi Ikeda, Hiroki Hata

**Affiliations:** a Department of Biology, Hirosaki University, Hirosaki, Japan; b Graduate School of Agriculture and Biosciences, Hirosaki University, Hirosaki, Japan; c Graduate School of Science and Engineering, Ehime University, Matsuyama, Japan

**Keywords:** *Microtus montebelli*, mitogenome, phylogenetic tree

## Abstract

The complete mitochondrial DNA sequence of the Japanese field vole *Microtus montebelli* was determined using Illumina MiSeq platform. The assembled genome was 16,307 bp in length and contained 13 protein-coding genes, two ribosomal RNA genes, 22 transfer RNA genes. According to phylogenetic analysis of 13 protein-coding genes, *M. montebelli* and other *Microtus* species consist of paraphyletic clades and *M. montebelli* is most closely related to *M. kikuchii*, a species endemic to Taiwan.

The Japanese field vole *Microtus montebelli* is endemic to Japan, inhabiting cultivated fields, meadows, conifer plantations, and riverbanks (Ohdachi et al. 2015). Although they have been recognized as a pest that eats the bark and roots of apple orchard trees in areas with heavy snowfall, they are a locally endangered species in Kyushu and urban areas in Honshu (Murano et al. 2019). This warrants the development of genetic markers with varying evolutionary rates to infer dispersal patterns and genetic population structure of *M. montebelli*.

The specimen of *M. montebelli* was collected from an apple orchard in Hirosaki, Japan (40°34′48″N, 140°25′18″E) on 13 May 2019, with permission from Aomori Prefecture (order number: 4153), following Hirosaki University guidelines for the ethical treatment of animals. The specimen was deposited in Hirosaki University (Dr. Atsushi Sogabe, e-mail: atsushi.sogabe@hirosaki-u.ac.jp) under the voucher number HUA1900522. The genomic DNA was extracted using a DNeasy Tissue and Blood Kit (Qiagen, Hilden, Germany). Whole-genome shotgun sequencing (2 × 300 bp) was performed using the Illumina MiSeq platform (Illumina, Hayward, CA), yielding 7,798,791 paired raw reads. Assembly was conducted *via* MITObim v1.9 (Hahn et al. 2013) using *M. arvalis* (GenBank accession number: MG_948434) as the reference. The assembled mitogenome sequence was annotated using the MITOS (Bernt et al. 2013).

The complete mitogenome of *M. montebelli* was 16,307 bp in length and contained 13 protein-coding genes (PCGs), two rRNA genes, 22 tRNA genes, one origin of L strand replication, and one control region. The gene arrangement of *M. motebelli* was identical to that of other species in Arvicolinae. All PCGs initiate with ATN codons (*ND1* began with ATA, and *ND2*, *ND3* and *ND5* began with ATT). TAA and TAG are the termination codon for most of the PCGs, whereas the incomplete stop codons (TA–) were used in *ND4* and *COX3*. The overall nucleotide composition was A (32.8%), C (27.5%), G (13.3%), and T (26.4%), indicating an obvious A/T skew (59.2%).

Bayesian inference (BI) and maximum-likelihood (ML) were used to reconstruct the phylogenetic trees based on 13 protein-coding genes from 25 species of Arvicolinae with the Chinese hamster *Cricetulus griseus* as an outgroup. Phylogenetic analysis was conducted with BEAST 1.10.4 (Suchard et al. 2018) and RAxML-NG 1.0.1 (Kozlov et al 2019) for BI and ML, respectively. BI and ML yielded identical phylogenetic trees (Figure 1). Results of phylogenetic tree analysis showed that genus *Microtus* are paraphyletic, sharing a common ancestor with genus *Lasiopodomys* and *Neodon*. Interestingly, we also identified that *M. montebelli* is a sister species of *M. kikuchii*, a species endemic to Taiwan.

**Figure 1.**
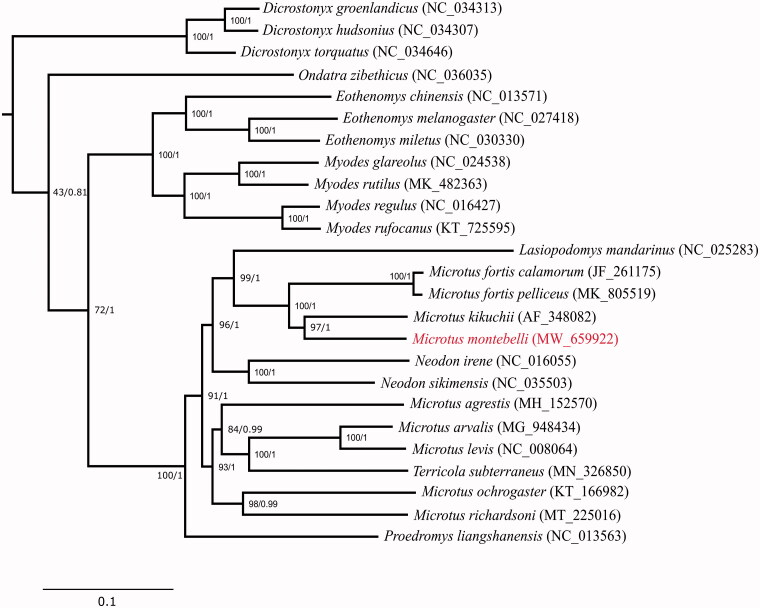
Maximum-likelihood tree of the subfamily Arvicolinae based on the sequences of 13 protein-coding genes (the outgroup is not shown for graphical reason). Number beside each node indicates bootstrap support values for ML (left) and posterior probabilities support values for BI (right).

## Data Availability

The data that support the findings of this study are available in NCBI at https://www.ncbi.nlm.nih.gov/, reference number MW659922.
